# Gluten‐Free Cakes With *Locusta migratoria* as a Protein Source: Volatile Profile, Quality, Nutritional, and Sensory Attributes

**DOI:** 10.1002/fsn3.72194

**Published:** 2026-07-29

**Authors:** Ceyda Dadalı, Yağmur Özcan

**Affiliations:** ^1^ Department of Food Engineering, Faculty of Engineering Ege University İzmir Türkiye

**Keywords:** defatted locust, gluten‐free cake, locust, *Locusta migratoria*, sustainability

## Abstract

The locust (*Locusta migratoria*), recognized as a sustainable alternative protein source, represents a promising ingredient for food enrichment. This study evaluated the effects of locust powder (LP) and defatted locust powder (DFLP), used as rice flour substitutes at 10%, 15%, and 20% levels, on volatile profile, quality, nutritional, microbiological, and sensory properties of gluten‐free (GF) cakes. GF cakes formulated with LP were designated as C10, C15, and C20, while those containing DFLP were labeled DFC10, DFC15, and DFC20. The incorporation of LP and DFLP significantly increased protein content of GF cakes, with the highest value observed in DFC20 sample (8.68 g/100 g) (*p* < 0.05). Total phenolic content and antioxidant activity were enhanced by both LP and DFLP; however, at equivalent substitution levels, DFLP containing cakes exhibited significantly higher values than LP containing cakes (*p* < 0.05). The specific volume of C10 and C20 samples was significantly higher than control (*p* < 0.05). The addition of LP and DFLP decreased L* and b* values and increased a* values of cake crumb (*p* < 0.05). DFC20 sample showed the highest hardness value (12.30 N). Both LP and DFLP increased the total essential and total non‐essential amino acid contents of the cakes (*p* < 0.05). Aldehydes were the dominant volatile compounds (127.83–46.18 mg/kg), followed by furans (17.26–51.06 mg/kg), and the highest aldehyde concentration was detected in C20 (*p* < 0.05). DFLP improved flavor properties more effectively than LP, while the overall acceptability of C10, C15, DFC10, and DFC15 samples was higher than control (*p* < 0.05). These results demonstrate the potential of LP and DFLP for improving GF bakery products while supporting sustainable food production.

## Graphical abstract

This study evaluated the effects of locust powder (LP) and defatted locust powder (DFLP), used as rice flour substitutes at 10%, 15%, and 20% levels, on volatile profile, quality, nutritional, microbiological, and sensory properties of gluten‐free (GF) cakes. GF cakes containing LP and DFLP exhibited higher ash, protein, total phenolic content, antioxidant activity, baking loss, and uniformity index values compared to the control sample, while specific volume and symmetry index values were reduced. The incorporation of LP and DFLP increased both the diversity and concentration of volatile compounds and improved the essential amino acid and essential fatty acid profiles of GF cakes.

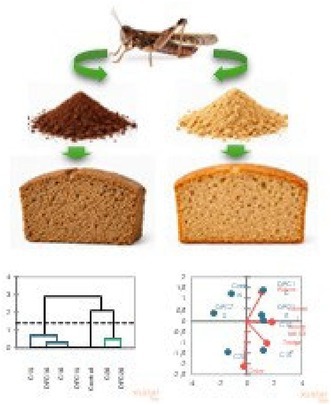

## 1 Introduction

Projections related to global population growth indicate that the number of people worldwide is expected to reach nearly nine billion by the middle of the century, with global food requirements increasing by approximately 70% (Gebreyes and Teka 2025). Furthermore, an increase in obesity and other metabolic diseases such as type 2 diabetes has been observed in recent years due to excessive food consumption (Ma et al. 2024). The increasing demand for food underlines the need to identify new approaches for sustainable food production. Within this framework, insects intended for human consumption are increasingly recognized as viable protein alternatives. To date, almost 2000 edible insects have been defined, including bees, wasps, ants, locusts, grasshoppers, and crickets (Kouřimská and Adámková 2016). The high nutritional value of edible insects, combined with lower environmental burden, supports their integration into future sustainable food systems (Lisboa et al. 2024; Gebreyes and Teka 2025). In contrast to traditional animal production systems, insect production requires markedly fewer natural resources and is associated with reduced greenhouse gas output (Skotnicka et al. 2021). Among edible insects, *Locusta migratoria* is particularly notable due to its high protein concentration, which reaches up to 71%, as well as its balanced essential amino acid composition and lipid fraction containing nutritionally valuable polyunsaturated fatty acids. These combined nutritional and functional characteristics suggest that locust‐derived ingredients could be effectively used for food product fortification (Clarkson et al. 2018; Brogan et al. 2021).

Recently, gluten‐free (GF) products consumption has increased on a global scale, driven by health‐related requirements and lifestyle choices. The decision to adopt a GF diet is influenced by various factors, including medical conditions such as coeliac disease, gluten sensitivity, as well as personal preferences (Mazzola et al. 2024; Zandonadi and Romão 2025). GF bakery products are generally prepared using starch‐rich flours such as rice, corn, potato, and bean, rather than gluten‐containing wheat flour (El Khoury et al. 2018). Nevertheless, the production of GF baked goods poses significant technological challenges and leads to nutritional deficiencies. GF products are characterized by low protein, high carbohydrate, and less desirable sensory properties (Vici et al. 2016). In order to surmount these limitations, innovative ingredients have been researched to improve GF product quality and nutritional profile.

Alternative protein sources incorporated into GF bakery products exhibit gluten‐like functionality, thereby masking the structural deficiencies typically observed in GF products (Moore et al. 2004). Additionally, previous studies have shown that utilizing protein sources in GF formulations can improve gas retention during baking, resulting in enhanced product volume, texture, and sensory quality (Wang et al. 2017; González et al. 2019). Previous studies using edible insects as an alternative protein source in GF products include the use of grasshopper powder in GF cookies (Özcan and Yılmaz 2025), cricket powder in GF bars (Pečová et al. 2023), cricket powder in GF crackers (Jirukkakul and Chanshotikul 2025), GF bread (Kowalczewski et al. 2021), and cricket powder in rice flour‐based cookies (Sanom and Jangchud 2020). These studies demonstrate a growing interest in the use of edible insects as an alternative protein source in GF products.

The application of *L. migratoria* as an alternative protein ingredient has been explored in various food products, including bread, muffins, crackers, three‐dimensional printed snacks, and ready‐to‐use therapeutic foods (Althwab et al. 2021; Çabuk 2021; Akande et al. 2023; Ivanišová et al. 2023; García‐Gutiérrez et al. 2024). Nevertheless, no published studies have specifically addressed the use of locust‐based ingredients in GF cake formulations. Due to the absence of a gluten network and the low protein content generally observed in GF cakes, the incorporation of locust powder (LP) and defatted locust powder (DFLP) is expected to improve the nutritional value of GF cakes; additionally, by enhancing batter stability and gas retention during baking, it may positively influence their physicochemical, textural, and sensory properties. Accordingly, the present research aimed to assess the suitability of LP and DFLP (*L. migratoria*) as sustainable protein alternatives by replacing rice flour at inclusion rates of 10%, 15%, and 20%, and to evaluate their influence on the physical, chemical, microbiological, and sensory attributes of GF cakes.

## 2 Material

All ingredients used in the preparation of GF cakes (rice flour, milk, egg, sunflower oil, sugar, and baking powder) were sourced from the local market (Migros, Türkiye). Locusts (*L. migratoria*) were supplied in frozen form by Mira Canlı Hayvan Böcek Turizm İnşaat Tarım Sanayi Ltd. Şti. (Antalya, Türkiye).

### 2.1 LP and DFLP Production

The locusts were subjected to a drying process in a tray dryer (Eksis, Türkiye) at 50°C with airflow set to 1.2 m/s. After drying, locusts were ground by a household grinder (Fakir, Germany) and subsequently passed through a 500 μm mesh to produce LP. To produce DFLP, a two‐stage extraction was performed using hexane (Bußler et al. 2016). The ground locusts and hexane were stirred for a period of one hour using a magnetic stirrer, with a ratio of 1:5 (w/v). Subsequently, the hexane–oil phase was separated, followed by a second extraction step using hexane. The hexane‐oil mixture was separated from the DFLP by decantation. Residual hexane was removed from DFLP by keeping it in an oven at 50°C overnight.

### 2.2 GF Cake Production

The cake formulation recommended by Gularte et al. (2012) was utilized in the production of the GF cakes. In the present study, LP and DFLF was used as a substitute for rice flour in GF cakes, at concentrations of 10%, 15%, and 20% (Table 1). The eggs and sugar were mixed with a household mixer for 1 min, after which other ingredients were added and mixed for a further 3 min. The prepared cake batter was transferred into a loaf pan and cooked in an oven (Bosch, Türkiye) at 170°C for 30 min. Following baking, GF cakes were allowed to cool at ambient conditions for 1 h and analyzed on the same day.

**TABLE 1:** GF cake ingredients.

GF cake ingredients (g)	Control	C10	C15	C20	DFC10	DFC15	DFC20
Rice flour	100.00	90.00	85.00	80.00	90.00	85.00	80.00
Locust flour	0.00	10.00	15.00	20.00	0.00	0.00	0.00
Defatted locust flour	0.00	0.00	0.00	0.00	10.00	15.00	20.00
Milk	75.00	75.00	75.00	75.00	75.00	75.00	75.00
Egg	62.50	62.50	62.50	62.50	62.50	62.50	62.50
Sunflower oil	37.50	37.50	37.50	37.50	37.50	37.50	37.50
Sugar	112.50	112.50	112.50	112.50	112.50	112.50	112.50
Baking powder	3.75	3.75	3.75	3.75	3.75	3.75	3.75

### 2.3 Composition Analysis

The moisture analyses of GF cake samples were implemented using AACC method 44‐15 (AACC 2000). The ash, fat, and protein contents were determined following AOAC methods 923.03, 945.16, and 955.04, respectively (AOAC 2007).

### 2.4 Color Analysis

The color properties of baked GF cakes were measured using a Konica Minolta CR‐400/410 colorimeter (Japan) under d/0 (diffuse illumination/0° viewing) geometry with illuminant C. The color values measured were determined as L* (0: black, 100: white), a* (+a: red, −a: green) and b* (+b: yellow, −b: blue) and recorded by direct contact with the surface of the cake samples. The total color change (∆*E*) was calculated using the $L_{c}^{*}$, $a_{c}^{*}$ and $b_{c}^{*}$ values of control GF cake (Equation 1).

(1) $$∆E=\sqrt{{\left(L^{*}-L_{c}^{*}\right)}^{2}+{\left(a^{*}-a_{c}^{*}\right)}^{2}+{\left(b^{*}-b_{c}^{*}\right)}^{2}}$$

### 2.5 Baking Loss

The calculation of baking loss was achieved by comparing the weight of GF cake batter prior to baking with the weight of baked GF cake that had been allowed to cool for 1 h. Baking loss values (%) were determined by measuring the difference between the weight of GF cake batter and the weight of baked GF cake, and then expressing this difference relative to the initial weight of batter (Martínez‐Cervera et al. 2015).

### 2.6 Specific Volume

The volume of GF cakes was measured using AACC method 10‐05.01 (2000). The specific volume was obtained by calculating the volume‐to‐weight ratio of the cake.

### 2.7 Symmetry and Uniformity Index

The structural characteristics of GF cakes were evaluated using the symmetry and uniformity index (AACC 2000). Following a cooling period of 1 h at ambient temperature, the baked GF cakes were bisected vertically through their centers. These samples were then utilized to calculate the symmetry index and uniformity index.

### 2.8 Texture Profile Analysis (TPA)

TPA of GF cake samples was performed to determine hardness, adhesiveness, springiness, cohesiveness, chewiness, and resilience using a texture analyzer (TA‐XT2, Stable Micro Systems, UK) fitted with a 36 mm cylindrical probe (P/36R). Prior to analysis, the samples were prepared with equal dimensions and a thickness of 2 cm. The compression test was conducted using a 25 kg load cell at 1 mm/s until 50% deformation of original height was achieved. The force–time curve was employed to calculate texture characteristics (Dadalı 2024).

### 2.9 Total Phenolic Content (TPC) and Antioxidant Activity (AA)

GF cakes were extracted with 80% methanol (v/v) to obtain extracts for TFC and AA analyses. For this purpose, 2.5 g of GF cake sample was extracted in a water bath set at 50°C (Memmert WNB, Germany) using 20 mL of 80% (v/v) methanol with continuous stirring at 150 rpm for 90 min. Following extraction, the mixture was centrifuged at 3400 g for 15 min (Hettich Universal 320, Germany). The supernatant was mixed with 80% (v/v) methanol to a final volume of 25 mL (Garcia‐Salas et al. 2010).

TPC was quantified using the Folin–Ciocalteu assay. Briefly, 50 μL of GF cake extract was mixed with 3 mL of distilled water and 250 μL of Folin–Ciocalteu reagent. Subsequently, 750 μL of 7% Na_2_CO_3_ solution was added, and the mixture was allowed to react for 8 min. Afterward, 950 μL of distilled water was added, and the samples were incubated in the dark for 2 h. Absorbance was measured at 765 nm using a spectrophotometer (Agilent Cary 60 UV–Vis, USA). Values were calculated from a gallic acid standard curve and expressed as milligrams of gallic acid equivalents (GAE) per gram of sample (Heimler et al. 2006).

The AA was measured using DPPH˙ assay and the percentage inhibition values obtained for the samples, in conjunction with a Trolox standard curve, were utilized to express the AA as μmol Trolox equivalents (TE)/g sample (Tomaino et al. 2010; Rapisarda et al. 2008). For this purpose, to determine AA, 1950 μL of DPPH˙ solution (100 μM) was added to 25, 50, and 75 μL of GF cake extract. It was then incubated for 20 min at room temperature. Subsequently, absorbance values were recorded at 517 nm using a spectrophotometer (Agilent Cary 60 UV–Vis, USA). Furthermore, ABTS˙+ radical cation decolorisation assay was conducted and reported as μmol TE/g of sample based on Trolox standard solutions, and percentage inhibition values (Re et al. 1999). A 7 mM ABTS˙+ solution containing 2.45 mM potassium persulfate was prepared, and 3960 μL of this solution was mixed with 20, 40, and 60 μL of GF cake extract. The mixtures were incubated in the dark for 6 min, and absorbance was measured at 734 nm using a spectrophotometer (Agilent Cary 60 UV–Vis, USA).

### 2.10 Amino Acid Analysis

For amino acid analysis, 0.1 g of each GF cake was hydrolyzed with 5 mL of 6 N HCl and kept at 110°C for 24 h. Following hydrolysis, the pH was adjusted to 6.7–7.3, after which the samples were centrifuged at 2700 g for 5 min and subsequently filtered. The amino acid profile was determined using an HPLC system (Shimadzu Nexera XR) with a Zorbax Eclipse AAA column (15 cm × 4.6 mm × 3.5 μm). Amino acids were identified by comparing retention times with external standards and quantified using calibration curves. The results were given as g/100 g of sample (Ata et al. 2024).

### 2.11 Volatile Compound Analysis

Headspace Solid Phase Microextraction (HS‐SPME) was utilized for extraction of volatile compounds from GF cakes. A total of 6 g of the homogenized sample was placed in a 40 mL vial and sealed with a Teflon‐coated silicone septum to extract volatile compounds. The prepared sample was then placed in a block heater at 60°C and allowed to equilibrate for 5 min. Then, a DVB/CAR/PDMS fiber was placed into the headspace, and extraction was continued for a duration of 60 min. After extraction, the SPME fiber was introduced into the GC–MS injection port (Hewlett‐Packard, 6890 GC–5973 MS) to thermally desorb the retained volatile compounds. Analyses were conducted in splitless mode with the injector temperature set to 250°C. Volatile compounds were separated using an HP‐5 MS capillary column (30 m × 0.25 mm i.d., 0.25 μm Agilent Technologies), with helium serving as the carrier gas at a constant flow rate of 1.0 mL/min. The oven temperature program began at 50°C, held for 5 min, then ramped to 220°C at 5°C/min and maintained for an additional 5 min (Garvey et al. 2021). The identification was implemented by Kovats index, utilizing the C7–C30 alkane set, with the Wiley and NIST libraries.

### 2.12 Fatty Acid Composition Analysis

The fatty acid composition of GF cakes was analyzed according to COI/T.20/Doc. No 33 (International Olive Council 2017). Fatty acids were identified using a 37‐component FAME standard mixture (Supelco, USA). Oil samples (0.1 g) were dissolved in 2 mL of heptane and transesterified with 0.2 mL of 2 M methanolic potassium hydroxide. The resulting methyl esters (1 μL) were analyzed by GC‐FID (Agilent 6890, USA) using an HP‐88 capillary column (60 m × 0.25 mm × 0.2 μm). The oven temperature was increased from 120°C to 240°C at a heating rate of 4°C/min, while the injector and detector were maintained at 250°C and 260°C, respectively. Individual fatty acids were identified by comparing their retention times with those of the 37‐component FAME standard mixture analyzed under identical chromatographic conditions. Fatty acids were determined based on relative peak area percentages.

### 2.13 Microbiological Analysis

GF cake samples were prepared according to ISO 6887‐1:2017 (ISO 2017). Ten grams of GF cake sample, with increased surface area through fragmentation, was weighed and transferred into a sterile stomacher bag containing 90 mL of sterile saline solution. The sample was homogenized for 3 min at 8 strokes/s using a BagMixer 400 W laboratory blender (Interscience, Saint Nom la Bretêche, France). Total aerobic mesophilic bacteria were enumerated according to ISO 4833‐1:2013. Appropriate serial dilutions were surface‐plated on Plate Count Agar (PCA) and incubated at 30°C for 72 h (ISO 2013). Yeasts and molds were enumerated following the general principles of ISO 21527‐2:2008. In brief, 1 mL aliquots of suitable decimal dilutions were inoculated onto Sabouraud dextrose agar supplemented with chloramphenicol (Biocorp). Plates were incubated at 25°C for 3–5 days (ISO 2008). To detect the presence of pathogens in GF cakes, the number of β‐glucuronidase‐positive *Escherichia coli* was determined according to ISO 16649‐2:2001 (ISO 2001) standard; the number of coagulase‐positive staphylococci ( *Staphylococcus aureus* and other staphylococci) was determined according to ISO 6888‐1:2021 (ISO 2021) standard; and the number of *Bacillus cereus* bacteria was determined according to ISO 7932:2004 (ISO 2004) method. All results were expressed as cfu/g GF cake.

### 2.14 Sensory Analysis

The sensory properties of GF cakes were assessed using a nine‐point hedonic scale, where scores ranged from 1 (dislike extremely) to 9 (like very much). A total of 54 panelists, aged between 18 and 53, participated in the sensory evaluation. GF cakes were presented to the panelists using randomly selected 3‐digit coded numbers. Panelists evaluated the samples based on color, texture, flavor, and overall acceptability (Altuğ Onoğur and Elmacı 2015). Ethical approval was obtained from the Scientific Research Ethics Committee of Ege University (Approval No. 2956).

### 2.15 Statistical Evaluation

Statistical analysis of the results was performed using the SPSS v.25 (IBM, USA) package program. Significant differences between GF cakes were determined using Variance Analysis and followed by Duncan's multiple range test at a significance level of 0.05. Cluster analysis and principal component analysis were conducted utilizing XLSTAT 2025.

## 3 Results and Discussion

### 3.1 Physical and Chemical Properties of GF Cakes

The physical and chemical properties of the GF cakes are presented in Table 2. Statistical analysis indicated that substituting LP and DFLP had no significant influence on moisture content (*p* > 0.05). Similarly, Bawa et al. (2020) determined that using cricket in bread formulations did not lead to a change in moisture content. In another study where cricket powder was added to GF bread, no statistically significant difference was found between the control group and GF bread with 10% and 20% cricket powder added (*p* > 0.05) (Da Rosa Machado and Thys 2019). Furthermore, in another study where mealworms were added to pancakes, while no statistically significant difference was found in moisture content with up to 20% mealworm addition (*p* > 0.05), a statistically significant difference was found in pancakes with 30% mealworm addition (*p* < 0.05) (Mazurek et al. 2023). These findings suggest that the addition of moderate levels of insect meal may not be sufficient to significantly alter the moisture retention properties of baked goods. Incorporation of LP and DFLP into GF cakes resulted in a significant increase in ash content (*p* < 0.05). The ash values of the samples ranged between 0.96 g/100 g and 1.18 g/100 g. Adding DFLP to GF cakes increased the ash content more than adding LP (*p* < 0.05). In a study where various insects and defatted insects were added to bread formulations, it was concluded that ash content was not affected by insect or defatting processes (Bottle et al. 2024). In another study, it was observed that adding cricket powder to bread formulations increased ash content, while adding it to cookie formulations did not lead to any change (Bawa et al. 2020). GF cookies with 5% grasshopper powder added were found to have an ash content of 1.62%. The addition of grasshopper powder was found to increase the ash content of the cookies compared to the control group (Özcan and Yılmaz 2025). In another study, it was determined that adding cricket powder to pancake at a rate of 20% or more increased the ash content. Pancake containing 20% cricket powder was found to have an ash content of 1.12% (Mazurek et al. 2023). The incorporation of LP and DFLP into GF cake formulations led to a statistically significant increase in protein content (*p* < 0.05). This increase can be attributed to the substantially higher protein content of locust flour compared to rice flour, resulting in protein enrichment of the cake formulations as the substitution level increased. Among the samples, C20 and DFC20 exhibited higher protein levels compared to the others, with values of 8.43 and 8.68 g/100 g, respectively (*p* < 0.05). However, defatting had no significant effect on the protein content of GF cakes (*p* > 0.05). Although defatting generally increases the relative proportion of protein in insect flour by removing lipids, no significant differences were observed between LP and DFLP containing cakes, suggesting that the effect was not sufficiently pronounced in the final product matrix. Indriani et al. (2020) reported a similar trend and significant enhancement in protein content in cakes fortified with increasing amounts of Bombay locust (*Patanga succincta* L.). In studies where insects such as mealworms, crickets, and buffalo worms were added to pancakes (Mazurek et al. 2023) and nut bars (Kowalski, Oracz, et al. 2022), samples containing insect substitutes were found to have higher protein content compared to the control group. The fat content of GF cakes ranged from 12.97 to 13.39 g/100 g. GF cakes with DFLP had a lower fat content than GF cakes with LP (*p* < 0.05). This result is directly associated with the removal of lipids during the defatting process, which reduced the amount of fat contributed by the insect flour to the final formulation. In parallel with this study, Bottle et al. (2024) revealed that defatting the insect species added to the bread formulation reduced the fat content of the breads. One study concluded that adding 5% mealworm, grasshopper, and cricket powder to crackers increased their fat content (Ivanišová et al. 2023). Another study found that adding mealworm, buffalo worm, and cricket powder to pancakes significantly increased the fat content of the insect‐added samples compared to the control group, especially at high substitution rates (Mazurek et al. 2023).

**TABLE 2:** Physical and chemical characteristics of GF cakes.^1^ ^,^ ^2^

Sample	Moisture (g/100 g)	Ash (g/100 g)	Protein (g/100 g)	Fat (g/100 g)	TPC (mg GAE/g)	AA with DPPH (μmol Trolox/g)	AA with ABTS (μmol Trolox/g)	Baking loss (%)	Specific volume (cm^3^/g)	Symmetry index (mm)	Uniformity index (mm)
Control	20.43 ± 1.05^a^	0.96 ± 0.03^a^	4.73 ± 0.21^a^	13.16 ± 0.21^ab^	0.12 ± 0.01^a^	0.64 ± 0.02^a^	1.18 ± 0.02^a^	8.60 ± 0.10^a^	2.27 ± 0.01^e^	10.03 ± 0.35^d^	0.03 ± 0.06^a^
C10	19.60 ± 1.35^a^	1.01 ± 0.05^b^	6.35 ± 0.12^b^	13.21 ± 0.02^ab^	0.47 ± 0.002^b^	1.71 ± 0.01^b^	3.49 ± 0.01^b^	8.87 ± 0.05^bc^	2.30 ± 0.01^f^	5.07 ± 0.21^b^	3.07 ± 0.25^c^
C15	19.38 ± 1.17^a^	1.04 ± 0.03^bc^	7.49 ± 0.16^c^	13.35 ± 0.24^b^	0.65 ± 0.002^c^	2.39 ± 0.03^d^	3.66 ± 0.01^c^	8.90 ± 0.12^c^	2.29 ± 0.02^f^	7.03 ± 0.12^c^	5.07 ± 0.06^e^
C20	18.94 ± 1.12^a^	1.07 ± 0.01^c^	8.43 ± 0.11^d^	13.39 ± 0.10^b^	0.80 ± 0.00^d^	2.52 ± 0.01^e^	4.35 ± 0.00^d^	8.93 ± 0.08^c^	2.16 ± 0.01^d^	1.01 ± 0.03^a^	3.03 ± 0.21^c^
DFC10	19.80 ± 1.52^a^	1.12 ± 0.02^d^	6.47 ± 0.19^b^	13.07 ± 0.02^a^	0.79 ± 0.013^d^	2.31 ± 0.01^c^	4.97 ± 0.01^e^	8.71 ± 0.08^ab^	2.12 ± 0.01^c^	15.07 ± 0.06^f^	4.97 ± 0.06^e^
DFC15	19.59 ± 0.35^a^	1.15 ± 0.01^de^	7.68 ± 0.08^c^	13.03 ± 0.03^a^	1.19 ± 0.017^e^	3.05 ± 0.01^f^	7.03 ± 0.01^f^	8.86 ± 0.12^bc^	2.09 ± 0.04^b^	19.03 ± 0.06^g^	1.03 ± 0.06^b^
DFC20	19.05 ± 1.59^a^	1.18 ± 0.01^e^	8.68 ± 0.07^d^	12.97 ± 0.05^a^	1.37 ± 0.052^f^	3.17 ± 0.01^g^	7.54 ± 0.00^g^	8.91 ± 0.10^c^	2.02 ± 0.01^a^	12.00 ± 0.02^e^	4.00 ± 0.10^d^

^1^ The analysis results were given as arithmetic mean ± standard deviation.

^2^ Different letters indicate statistical differences within column (*p* < 0.05).

TPC of GF cakes was influenced by LP and DFLP replacement ratio (*p* < 0.05). TPC of samples was in the range 0.12–1.37 mg GAE/g. GF cakes containing DFLP were found to have a higher TPC compared to GF cakes containing LP (*p* < 0.05). DFC20 sample (1.37 mg GAE/g) had the highest TPC (*p* < 0.05). In a study where various insects were added to the bar formulation at rates of 15% and 30%, it was determined that insects led to an increase in TPC (Gumul et al. 2023). In a study where house cricket powder was added to GF bars, it was determined that the addition of house cricket powder increased the TPC of the bars compared to the control group (Pečová et al. 2023). *Locusta migratoria* was reported to contain various phenolic compounds, including anthocyanins, flavan‐3‐ols, flavonols, flavones, phenolic acids, and stilbenes (Rocchetti et al. 2024). LP and DFLP enrichment in GF cake formulation significantly affected AA of GF cake samples (*p* < 0.05). According to DPPH and ABTS analysis results, GF cake samples containing LP and DFLP showed higher AA (*p* < 0.05). According to DPPH and ABTS results, GF cakes containing DFLP were found to have higher AA compared to those containing LP (*p* < 0.05). DFC20 sample exhibited significantly higher antioxidant activity compared to the other GF cakes, based on DPPH and ABTS assay results of 3.17 and 7.54 μmol Trolox/g, respectively. Similarly, Kowalski et al. (2023) reported that the use of buffalo worm, cricket, and mealworm increased the AA of sponge cakes (*p* < 0.05). In a study where cricket powder was added to GF bars, the AA of the bars with added cricket powder, as determined by DPPH and ABTS methods, was found to be significantly higher than that of the control group. Specifically, GF bars containing 25% cricket powder had approximately 7–8 times higher AA than the control group (Pečová et al. 2023).

The physical analysis results of GF cake samples showed that increasing the addition of LP and DFLP resulted in increased baking loss (*p* < 0.05). Similarly, it has been reported that adding insect meal to sponge cakes increases baking loss compared to the control group (Talens et al. 2022). Specific volume values differed significantly among GF cake samples (*p* < 0.05). It was determined that adding LP to GF cakes resulted in a higher specific volume compared to adding DFLP (*p* < 0.05). Moreover, higher levels of LP and DFLP incorporation were associated with a reduction in specific volume. The lowest specific volume was found in the DFC20 sample (2.02 cm^3^/g) (*p* < 0.05). The specific volumes of C10 and C15 were higher than other GF cakes (*p* < 0.05). Indriani et al. (2020) prepared cakes with different levels of Bombay locust and observed a decrease in specific volume with increasing Bombay locust addition. In the cake enriched with 30% Bombay locust, the specific volume was reported to be 2.76 cm^3^/g. In another study, it was determined that when cricket flour was added to sponge cake, the specific volume of cakes with added cricket flour decreased compared to the control group (Vlahova‐Vangelova et al. 2022). Surface contour characteristics were assessed using the symmetry index, while overall cake symmetry was evaluated through the uniformity index. Statistical analysis showed that GF cakes differed significantly with respect to both indices (*p* < 0.05). As the addition levels of LP and DFLP increased, the symmetry index of GF cakes decreased (*p* < 0.05). The lowest symmetry index was observed in the C20 sample (1.01 mm) (*p* < 0.05). The symmetry index of GF cakes supplemented with DFLP was found to be higher compared to GF cakes supplemented with LP (*p* < 0.05). In cakes, it is important that the batter develops symmetrically during baking, forming a peak in the center. Therefore, cakes with a symmetry index greater than zero are considered desirable. Lower symmetry index values reflect the formation of a flatter cake surface (Felisberto et al. 2015). Analysis of the uniformity index showed that the control sample exhibited the minimum value (0.03 mm) (*p* < 0.05). The uniformity index increased significantly with the addition of LP and DFLP (*p* < 0.05). The uniformity index is determined by measuring the height difference between the two ends of the cake samples. Values closer to zero indicate uniform dough expansion and better preservation of cake structure (Kılıç and Boz 2025). Similarly, Indriani et al. (2020) observed a symmetry index decrease with the addition of Bombay locust to cake formulations. Furthermore, an increase in the uniformity index was found particularly at substitution levels greater than 10%, compared to the control sample.

### 3.2 Color Properties of GF Cakes

Color properties, namely L*, a*, b*, and Δ*E* values of GF cake samples are presented in Table 3. Varying the proportions of LP and DFLP in GF cake formulations significantly affected the L*, a*, and b* color parameters (*p* < 0.05). The control sample exhibited higher lightness values in both crumb and crust measurements (*p* < 0.05). A significant decline in L* was observed with increasing levels of LP and DFLP use in GF cakes (*p* < 0.05). It was determined that adding DFLP to GF cakes resulted in higher L* values compared to adding LP (*p* < 0.05). According to crust color characteristics, the redness decreased as the addition levels of LP and DFLP increased (*p* < 0.05). Crumb color evaluation revealed that the incorporation of LP and DFLP resulted in a statistically significant rise in a* values relative to control sample (*p* < 0.05). Higher levels of LP and DFLP incorporation were associated with a significant increase in a* values of the cake crumb (*p* < 0.05). It was found that the addition of DFLP decreased a* values compared to the addition of LP (*p* < 0.05). Incorporation of LP and DFLP into GF cakes resulted in a significant reduction in yellowness values of both crust and crumb (*p* < 0.05). When DFLP was added to GF cakes, they were found to have higher b* values than when LP was added to GF cakes (*p* < 0.05). Δ*E*, known as total color difference, is used to describe color change. A progressive rise in Δ*E* values was noted as the proportion of LP and DFLP in the formulations increased. A Δ*E* value higher than 12.0 indicates a very evident color difference (Vurro et al. 2022). In samples containing LP and DFLP, Δ*E* values were greater than 12.0 in both the crumb and crust. Therefore, it was concluded that significant differences existed in the color characteristics of the samples. The difference in color values between GF cakes containing LP and DFLP and the control sample can be explained by the color of the locust used. *Locusta migratoria* contains various carotenoids, such as β‐carotene, β‐cryptoxanthin, α‐carotene, lutein, zeaxanthin, and astaxanthin, depending on its food source and diet (Barutçu Mazı et al. 2025). These pigments, which determine the characteristic color of the locust, are lipophilic and soluble in the lipid phase. Therefore, the partial removal of these lipophilic pigments during the fat removal process can affect the color parameters of the product (Spindola Marasca et al. 2025). In this study, lower a* and Δ*E* values were observed in cakes containing DFLP compared to cakes containing LP. This observation can be explained by the reduced contribution of fat‐soluble color pigments to the cake color as a result of their partial removal from the locust during the fat removal process.

**TABLE 3:** Color properties of GF cakes.^1^ ^,^ ^2^

	Crust color				Crumb color			
	L*	a*	b*	Δ*E*	L*	a*	b*	Δ*E*
Control	67.23 ± 0.52^f^	11.62 ± 0.19^cd^	29.68 ± 0.51^f^	—	78.31 ± 1.22^e^	−1.63 ± 0.23^a^	22.30 ± 0.56^e^	—
C10	44.75 ± 0.81^c^	12.46 ± 0.21^e^	14.58 ± 0.68^c^	27.09	44.62 ± 0.71^c^	8.33 ± 0.11^c^	17.45 ± 1.01^d^	35.47
C15	40.96 ± 0.46^b^	11.92 ± 0.19^d^	12.04 ± 0.36^b^	31.64	39.61 ± 0.83^b^	9.24 ± 0.19^de^	12.75 ± 0.54^b^	41.32
C20	39.68 ± 0.28^a^	11.58 ± 0.32^c^	10.47 ± 0.46^a^	33.59	37.71 ± 0.99^a^	9.77 ± 0.31^f^	10.47 ± 1.26^a^	43.80
DFC10	54.82 ± 0.37^e^	11.78 ± 0.11^cd^	23.95 ± 0.23^e^	13.67	50.69 ± 0.20^d^	7.85 ± 0.14^b^	18.53 ± 0.42^d^	29.44
DFC15	49.02 ± 0.45^d^	11.06 ± 0.11^b^	18.87 ± 0.24^d^	21.18	44.06 ± 0.43^c^	8.94 ± 0.21^d^	15.25 ± 0.61^c^	36.53
DFC20	41.35 ± 0.38^b^	10.48 ± 0.22^a^	14.90 ± 0.28^c^	29.82	38.04 ± 0.39^a^	9.27 ± 0.18^e^	11.69 ± 0.44^b^	43.05

^1^ The analysis results were given as arithmetic mean ± standard deviation.

^2^ Different letters indicate statistical differences within column (*p* < 0.05).

### 3.3 Textural Properties of GF Cakes

Texture analysis results are given in Table 4. When the texture analysis results were examined, statistically significant differences were found in terms of hardness among the GF cake samples (*p* < 0.05). The hardness of DFC20 (12.36 N) was higher than other GF cakes (*p* < 0.05). The increased hardness observed in DFC20 may be attributed to the higher protein concentration and lower fat content of DFLP. Fat generally contributes to tenderness in cake systems, whereas protein‐rich ingredients may promote the formation of a denser crumb structure, resulting in increased hardness. In a study examining pancakes enriched with *Acheta domesticus* , the sample containing 30% cricket flour was found to have the highest hardness (Bruttomesso et al. 2024). Xie et al. (2022) found that adding mealworm powder to bread did not affect hardness at substitution rates up to 10%, but hardness increased at a 15% substitution level. The adhesiveness values of GF cakes decreased significantly compared to the control sample with the addition of LP and DFLP to the formulation (*p* < 0.05). This indicates that adding a high amount of DFLP reduces the adhesiveness of the cake. Springiness values of C20 were significantly lower than the control sample (*p* < 0.05). As the amount of locust substitute increased, the springiness values decreased, indicating a reduction in elastic recovery after compression. This can be explained by the degradation of the starch‐based structure in GF cakes. A study on brownies enriched with cricket powder found that the addition of cricket powder reduced springiness compared to the control (Ho et al. 2022). Cohesiveness values did not differ significantly among the GF cake samples (*p* > 0.05). Similarly, a study by Pauter et al. (2018) reported that adding cricket powder to muffins did not statistically affect cohesiveness values (*p* > 0.05). Chewiness values were significantly lower in all LP and DFLP containing GF cakes relative to the control (*p* < 0.05). These findings suggest that incorporating LP and DFLP into GF cake leads to a reduction in cake chewiness. Specifically, the GF cake with 20% LP added showed the lowest chewiness (0.37). This reduction is consistent with the combined decrease in hardness and springiness, suggesting a weaker and less resistant cake structure. The incorporation of locust flour may have produced a softer matrix with reduced resistance to mastication, likely due to changes in protein–starch interactions and internal crumb architecture. It was reported that adding insect flour to sponge cakes reduces chewiness (Talens et al. 2022). A similar effect was observed in brownies enriched with cricket powder (Ho et al. 2022). Resilience analysis showed a significant reduction following the incorporation of LP into the cake formulation compared with the control (*p* < 0.05). Among the samples, C20 and DFC20 exhibited the lowest resilience values (0.06). The reduction in resilience indicates a diminished ability of the cake structure to recover after deformation. This may be associated with weakened internal network formation in GF systems upon substitution with insect flour, which reduces the elasticity and structural integrity of the crumb. Similarly, it has been reported that adding mealworm and cricket powder to muffins reduces their resilience (Zielińska et al. 2021; Pauter et al. 2018).

**TABLE 4:** Textural properties of GF cakes.^1^ ^,^ ^2^

	Hardness (N)	Adhesiveness (g·s)	Springiness	Cohesiveness	Chewiness	Resilience
Control	10.01 ± 1.67^a^	−0.16 ± 0.08^d^	0.27 ± 0.05^bc^	0.26 ± 0.03^a^	1.03 ± 0.17^c^	0.10 ± 0.02^d^
C10	9.81 ± 0.34^a^	−0.40 ± 0.05^cd^	0.24 ± 0.02^bc^	0.23 ± 0.01^a^	0.75 ± 0.07^b^	0.07 ± 0.00^abc^
C15	10.85 ± 0.79^ab^	−0.66 ± 0.04^c^	0.22 ± 0.03^ab^	0.20 ± 0.01^a^	0.65 ± 0.04^b^	0.06 ± 0.01^ab^
C20	10.60 ± 0.75^a^	−0.69 ± 0.09^c^	0.20 ± 0.01^a^	0.17 ± 0.02^a^	0.37 ± 0.08^a^	0.05 ± 0.01^a^
DFC10	10.18 ± 0.85^a^	−0.72 ± 0.15^c^	0.28 ± 0.00^c^	0.28 ± 0.02^a^	0.77 ± 0.07^b^	0.09 ± 0.01^cd^
DFC15	11.22 ± 0.26^ab^	−1.12 ± 0.38^b^	0.26 ± 0.02^bc^	0.26 ± 0.11^a^	0.61 ± 0.18^b^	0.08 ± 0.03^bcd^
DFC20	12.36 ± 0.93^b^	−3.03 ± 0.21^a^	0.24 ± 0.01^bc^	0.20 ± 0.01^a^	0.65 ± 0.02^b^	0.06 ± 0.00^ab^

^1^ The analysis results were given as arithmetic mean ± standard deviation.

^2^ Different letters indicate statistical differences within column (*p* < 0.05).

Low hardness and chewiness are desirable characteristics in GF cakes. It is known that the lower these parameters, the softer the cake (Li et al. 2020). In this study, sample C20, in particular, showed lowest chewiness (*p* < 0.05) and statistically the same hardness as the control GF cake. It is thought that the GF cake with added 20% LP (C20), which has similar hardness to the control sample but lower chewiness, exhibits positive textural characteristics.

### 3.4 Amino Acid Profile of GF Cakes

Eighteen amino acids were detected in the GF cake samples, comprising nine essential and nine non‐essential amino acids, as summarized in Table 5. Consistent with these findings, Brogan et al. (2021) reported an identical distribution of essential and non‐essential amino acids in *L. migratoria*. Statistical analysis revealed significant variations among the GF cake formulations for all identified amino acids (*p* < 0.05). The addition of LP or DFLP to the GF cakes resulted in an increase in all amino acids except cysteine (*p* < 0.05). Furthermore, as the percentage of LP or DFLP added increased, an increase was detected in all essential and non‐essential amino acids except cysteine and tryptophan in GF cakes (*p* < 0.05). Adding 15% LP and DFLP to GF cake formulation resulted in a statistically significant increase in cysteine, a non‐essential amino acid, and tryptophan, an essential amino acid (*p* < 0.05). The removal of locust oil did not cause significant changes in histidine, tryptophan, or cysteine levels in GF cakes containing the same amounts of LP and DFLP (*p* > 0.05). No statistically significant variations in lysine, threonine, valine, alanine, aspartic acid, or serine were found between C10 and DFC10 samples at a 10% substitution level (*p* > 0.05), whereas significant differences emerged between C15 and DFC15 samples when the level was raised to 15% (*p* < 0.05). The isoleucine, leucine, methionine, phenylalanine, arginine, glutamic acid, proline, and tyrosine contents of GF cakes with added LP were found to be lower than that of GF cakes with added DFLP (*p* < 0.05). Total essential amino acid (TEAA), Total non‐essential amino acid TNEAA, and total amino acid (TAA) values differed significantly among GF cakes (*p* < 0.05), with LP and DFLP fortified cakes showing increased amino acid contents (*p* < 0.05). TEAA, TNEAA, and TAA contents of GF cakes containing LP were lower compared to samples containing DFLP (*p* < 0.05). DFC20 sample exhibited the greatest TEAAC value, reaching 2.71 g/100 g. Bottle et al. (2024) revealed TEAA content increase in breads when insect species were added to the bread formulation. Furthermore, breads produced using defatted insects showed an increase in TEAA content compared to breads prepared with insects. The addition of LP to ready‐to‐use therapeutic foods resulted in an increase in all profiled amino acids (Akande et al. 2023). This study showed that LP and DFLP had a significant effect on amino acid levels. Adding LP and DFLP to the formulation enriched GF cakes with TEAA and TNEAA. It is thought that LP and DFLP could be a good source, especially for essential amino acids, which cannot be synthesized in the body and therefore must be obtained through diet.

**TABLE 5:** Amino acid profile of GF cakes (g/100 g).^1^ ^,^ ^2^

	Control	C10	C15	C20	DFC10	DFC15	DFC20
Essential amino acids							
Histidine	0.09 ± 0.00^a^	0.13 ± 0.00^b^	0.15 ± 0.00^c^	0.17 ± 0.00^d^	0.13 ± 0.00^b^	0.15 ± 0.00^c^	0.17 ± 0.00^d^
Isoleucine	0.15 ± 0.01^a^	0.22 ± 0.01^b^	0.26 ± 0.01^d^	0.29 ± 0.00^e^	0.23 ± 0.01^c^	0.26 ± 0.00^d^	0.30 ± 0.00^f^
Leucine	0.29 ± 0.00^a^	0.41 ± 0.01^b^	0.47 ± 0.00^d^	0.54 ± 0.00^f^	0.42 ± 0.00^c^	0.49 ± 0.01^e^	0.55 ± 0.01^g^
Lysine	0.27 ± 0.00^a^	0.36 ± 0.01^b^	0.40 ± 0.00^c^	0.45 ± 0.00^e^	0.36 ± 0.00^b^	0.41 ± 0.00^d^	0.46 ± 0.01^f^
Methionine	0.09 ± 0.00^a^	0.11 ± 0.00^b^	0.12 ± 0.00^cd^	0.13 ± 0.01^e^	0.12 ± 0.00^c^	0.13 ± 0.00^de^	0.14 ± 0.00^f^
Phenylalanine	0.19 ± 0.01^a^	0.23 ± 0.01^b^	0.25 ± 0.01^d^	0.28 ± 0.01^f^	0.24 ± 0.00^c^	0.26 ± 0.00^e^	0.29 ± 0.00^g^
Threonine	0.21 ± 0.00^a^	0.27 ± 0.00^b^	0.29 ± 0.01^c^	0.32 ± 0.00^e^	0.27 ± 0.01^b^	0.30 ± 0.01^d^	0.33 ± 0.00^f^
Tryptophan	0.03 ± 0.00^a^	0.05 ± 0.00^b^	0.05 ± 0.00^b^	0.06 ± 0.00^c^	0.05 ± 0.01^b^	0.05 ± 0.00^b^	0.06 ± 0.00^c^
Valine	0.19 ± 0.00^a^	0.29 ± 0.00^b^	0.34 ± 0.00^c^	0.39 ± 0.01^e^	0.29 ± 0.00^b^	0.35 ± 0.00^d^	0.40 ± 0.00^f^
Non‐essential amino acids							
Alanine	0.16 ± 0.00^a^	0.35 ± 0.01^b^	0.44 ± 0.012^c^	0.54 ± 0.01^e^	0.36 ± 0.01^b^	0.46 ± 0.01^d^	0.56 ± 0.01^f^
Arginine	0.23 ± 0.00^a^	0.32 ± 0.01^b^	0.36 ± 0.004^d^	0.41 ± 0.01^f^	0.33 ± 0.00^c^	0.38 ± 0.00^e^	0.42 ± 0.00^g^
Aspartic acid	0.33 ± 0.01^a^	0.44 ± 0.00^b^	0.48 ± 0.010^c^	0.55 ± 0.01^e^	0.44 ± 0.00^b^	0.50 ± 0.01^d^	0.56 ± 0.00^e^
Cysteine	0.12 ± 0.00^a^	0.12 ± 0.00^a^	0.13 ± 0.003^b^	0.13 ± 0.00^b^	0.12 ± 0.00^a^	0.13 ± 0.00^b^	0.13 ± 0.00^b^
Glutamic acid	0.53 ± 0.01^a^	0.67 ± 0.01^b^	0.72 ± 0.002^d^	0.81 ± 0.01^f^	0.68 ± 0.00^c^	0.75 ± 0.00^e^	0.82 ± 0.00^g^
Glycine	0.13 ± 0.00^a^	0.23 ± 0.001^b^	0.27 ± 0.003^d^	0.33 ± 0.00^f^	0.24 ± 0.00^c^	0.29 ± 0.00^e^	0.34 ± 0.00^g^
Proline	0.20 ± 0.00^a^	0.30 ± 0.00^b^	0.35 ± 0.003^d^	0.40 ± 0.00^f^	0.31 ± 0.01^c^	0.36 ± 0.00^e^	0.42 ± 0.00^g^
Serine	0.23 ± 0.00^a^	0.28 ± 0.00^b^	0.30 ± 0.003^c^	0.33 ± 0.01^e^	0.28 ± 0.00^b^	0.31 ± 0.00^d^	0.34 ± 0.00^f^
Tyrosine	0.16 ± 0.00^a^	0.25 ± 0.00^b^	0.29 ± 0.001^d^	0.34 ± 0.00^f^	0.26 ± 0.00^c^	0.30 ± 0.00^e^	0.35 ± 0.00^g^
Nutritional qualities							
TEAA	1.53 ± 0.01^a^	2.07 ± 0.01^b^	2.34 ± 0.02^d^	2.63 ± 0.02^f^	2.12 ± 0.02^c^	2.42 ± 0.01^e^	2.71 ± 0.01^g^
TNEAA	2.10 ± 0.02^a^	2.96 ± 0.01^b^	3.36 ± 0.02^d^	3.85 ± 0.01^f^	3.03 ± 0.02^c^	3.49 ± 0.01^e^	3.95 ± 0.01^g^
TAA	3.64 ± 0.03^a^	5.04 ± 0.0^b^	5.71 ± 0.03^d^	6.48 ± 0.01^f^	5.15 ± 0.02^c^	5.90 ± 0.01^e^	6.66 ± 0.02^g^

^1^ The analysis results were given as arithmetic mean ± standard deviation.

^2^ Different letters indicate statistical differences within row (*p* < 0.05).

### 3.5 Fatty Acid Profile of GF Cakes

The fatty acid composition of the GF cake samples is presented in Table 6. The detected fatty acids ranged in chain length from 4 to 24 carbon atoms. No statistically significant differences were observed among all samples in terms of butyric acid, caproic acid, caprylic acid, capric acid, myristic acid, myristoleic acid, palmitic acid, palmitoleic acid, heptadecanoic acid, stearic acid, oleic acid, linoleic acid, arachidic acid, eicosenoic acid, behenic acid, erucic acid, and lignoceric acid (*p* > 0.05). A statistically significant difference was observed in α‐linolenic acid content among all samples (*p* < 0.05). The highest amount of α‐linolenic acid was determined in sample C20 (0.53%), while the lowest amount was found in the control sample (0.16%). An increase in α‐linolenic acid was observed proportionally with the addition of LP. The addition of 20% DFLP resulted in a higher amount of α‐linolenic acid compared to the control sample. Previous studies have reported that locusts contain essential fatty acids associated with various health benefits. In a study investigating various characteristics of *L. migratoria*, Clarkson et al. (2018) reported that this locust species contains high levels of oleic acid and palmitic acid.

**TABLE 6:** Fatty acid profile of GF cakes (%).^1^ ^,^ ^2^

	Control	C10	C15	C20	DFC10	DFC15	DFC20
Butyric acid (C4:0)	0.12 ± 0.01^a^	0.12 ± 0.01^a^	0.12 ± 0.01^a^	0.12 ± 0.01^a^	0.12 ± 0.01^a^	0.12 ± 0.01^a^	0.12 ± 0.01^a^
Caproic acid (C6:0)	0.09 ± 0.00^a^	0.08 ± 0.00^a^	0.08 ± 0.00^a^	0.08 ± 0.00^a^	0.09 ± 0.00^a^	0.09 ± 0.00^a^	0.09 ± 0.00^a^
Caprylic acid (C8:0)	0.06 ± 0.00^a^	0.06 ± 0.00^a^	0.06 ± 0.00^a^	0.06 ± 0.00^a^	0.06 ± 0.00^a^	0.06 ± 0.00^a^	0.06 ± 0.00^a^
Capric acid (C10:0)	0.14 ± 0.00^a^	0.14 ± 0.00^a^	0.14 ± 0.00^a^	0.14 ± 0.00^a^	0.14 ± 0.00^a^	0.14 ± 0.00^a^	0.14 ± 0.00^a^
Myristic acid (C14:0)	0.68 ± 0.03^a^	0.71 ± 0.04^a^	0.72 ± 0.04^a^	0.73 ± 0.04^a^	0.69 ± 0.03^a^	0.69 ± 0.04^a^	0.69 ± 0.03^a^
Myristoleic acid (C14:1)	0.02 ± 0.00^a^	0.02 ± 0.00^a^	0.02 ± 0.00^a^	0.02 ± 0.00^a^	0.02 ± 0.00^a^	0.02 ± 0.00^a^	0.02 ± 0.00^a^
Palmitic acid (C16:0)	9.45 ± 0.33^a^	9.67 ± 0.48^a^	9.78 ± 0.49^a^	9.88 ± 0.49^a^	9.47 ± 0.47^a^	9.48 ± 0.47^a^	9.50 ± 0.48^a^
Palmitoleic acid (C16:1)	0.62 ± 0.01^a^	0.63 ± 0.03^a^	0.63 ± 0.02^a^	0.64 ± 0.02^a^	0.62 ± 0.03^a^	0.63 ± 0.03^a^	0.63 ± 0.02^a^
Heptadecanoic acid (C17:0)	0.01 ± 0.00^a^	0.01 ± 0.00^a^	0.01 ± 0.00^a^	0.01 ± 0.00^a^	0.01 ± 0.00^a^	0.01 ± 0.00^a^	0.01 ± 0.00^a^
Stearic acid (C18:0)	4.06 ± 0.01^a^	4.10 ± 0.01^a^	4.12 ± 0.00^a^	4.14 ± 0.03^a^	4.07 ± 0.01^a^	4.07 ± 0.00^a^	4.07 ± 0.00^a^
Oleic acid (C18:1 cis)	33.32 ± 0.68^a^	33.37 ± 0.59^a^	33.39 ± 0.67^a^	33.41 ± 0.32^a^	33.33 ± 0.69^a^	33.33 ± 0.53^a^	33.33 ± 0.67^a^
Linoleic acid (C18:2 cis)	49.89 ± 1.09^a^	49.39 ± 0.97^a^	49.14 ± 1.06^a^	48.90 ± 1.45^a^	49.84 ± 0.49^a^	49.81 ± 1.19^a^	49.79 ± 0.79^a^
Arachidic acid (C20:0)	0.25 ± 0.01^a^	0.24 ± 0.01^a^	0.24 ± 0.01^a^	0.24 ± 0.01^a^	0.25 ± 0.01^a^	0.25 ± 0.01^a^	0.25 ± 0.01^a^
Eicosenoic acid (C20:1)	0.21 ± 0.01^a^	0.21 ± 0.01^a^	0.21 ± 0.01^a^	0.21 ± 0.01^a^	0.21 ± 0.01^a^	0.21 ± 0.01^a^	0.21 ± 0.01^a^
α‐linolenic acid (C18:3 n‐3)	0.16 ± 0.01^a^	0.34 ± 0.02^c^	0.44 ± 0.01^d^	0.53 ± 0.03^e^	0.17 ± 0.01^ab^	0.18 ± 0.02^ab^	0.19 ± 0.01^b^
Behenic acid (C22:0)	0.64 ± 0.03^a^	0.63 ± 0.03^a^	0.63 ± 0.03^a^	0.63 ± 0.03^a^	0.64 ± 0.03^a^	0.64 ± 0.03^a^	0.64 ± 0.03^a^
Erucic acid (C22:1)	0.03 ± 0.00^a^	0.03 ± 0.00^a^	0.03 ± 0.00^a^	0.03 ± 0.00^a^	0.03 ± 0.00^a^	0.03 ± 0.00^a^	0.03 ± 0.00^a^
Lignoceric acid (C24:0)	0.23 ± 0.01^a^	0.23 ± 0.01^a^	0.23 ± 0.01^a^	0.23 ± 0.01^a^	0.23 ± 0.01^a^	0.23 ± 0.01^a^	0.23 ± 0.01^a^
Index							
Total SFA	15.73 ± 0.14^a^	15.99 ± 0.79^a^	16.13 ± 0.75^a^	16.26 ± 0.59^a^	15.77 ± 0.80^a^	15.77 ± 0.61^a^	15.80 ± 0.93^a^
Total MUFA	34.20 ± 0.70^a^	34.26 ± 0.58^a^	34.28 ± 0.31^a^	34.31 ± 0.48^a^	34.21 ± 0.71^a^	34.22 ± 0.43^a^	34.22 ± 0.64^a^
Total PUFA	50.05 ± 1.42^a^	49.73 ± 1.30^a^	49.58 ± 1.41^a^	49.43 ± 1.56^a^	50.01 ± 1.37^a^	49.99 ± 1.13^a^	49.98 ± 1.27^a^
n‐3	0.16 ± 0.01^a^	0.34 ± 0.02^c^	0.44 ± 0.01^d^	0.53 ± 0.03^e^	0.17 ± 0.01^ab^	0.18 ± 0.02^ab^	0.19 ± 0.01^b^
n‐6	49.89 ± 1.09^a^	49.39 ± 0.97^a^	49.14 ± 1.06^a^	48.90 ± 1.45^a^	49.84 ± 0.49^a^	49.81 ± 1.19^a^	49.79 ± 0.79^a^
n‐6/n‐3	319.97 ± 1.82^g^	145.97 ± 0.90^c^	111.82 ± 1.27^b^	93.02 ± 0.79^a^	299.52 ± 2.01^f^	273.39 ± 1.43^e^	266.76 ± 1.68^d^

^1^ The analysis results were given as arithmetic mean ± standard deviation.

^2^ Different letters indicate statistical differences within row (*p* < 0.05).

There was no statistically significant difference between all samples in terms of total saturated fatty acids (SFA), total monounsaturated fatty acids (MUFA), and total polyunsaturated fatty acids (PUFA) (*p* > 0.05). In a study, it was reported that as the insect meal addition rate increased, the levels of total SFA and total MUFA increased, while the percentage of total PUFA decreased (Roncolini et al. 2020). Omega‐3 (n‐3) fatty acids exhibit anti‐inflammatory and cardioprotective effects, whereas omega‐6 (n‐6) fatty acids, although essential, may promote inflammation when consumed excessively; therefore, maintaining an appropriate balance is crucial (Simopoulos 2002). A balanced intake of n‐6 and n‐3 fatty acids is crucial for human health. A high n‐6/n‐3 ratio has been associated with overweight and obesity, whereas a lower and more balanced ratio is linked to reduced weight gain and improved metabolic health (Jeong et al. 2024). In this study, a decrease in the n‐6/n‐3 ratio was observed compared to the control sample as the LP and DFLP addition rates increased (*p* < 0.05). Specifically, the n‐6/n‐3 ratio of sample C20 is significantly lower compared to the control sample (*p* < 0.05). Increasing the LP supplementation rate has been found to improve the anti‐inflammatory and cardioprotective effects of n‐3 fatty acids. Kowalski et al. (2023) reported that the n‐6/n‐3 ratio increased as the addition rate of insect meal increased in sponge cakes. Although LP and DFLP addition significantly reduced the n‐6/n‐3 ratio relative to the control (*p* < 0.05), the values remained relatively high across all formulations. This is mainly attributed to the use of sunflower oil in the cake formulation, which is naturally rich in linoleic acid (n‐6). Future studies could improve the nutritional profile of these products by replacing sunflower oil with oils characterized by a more favorable fatty acid composition, such as canola (rapeseed) oil, flaxseed oil, or optimized vegetable oil blends, while maintaining the desired technological and sensory properties. Such modifications may further contribute to lowering the n‐6/n‐3 ratio of the final product.

### 3.6 Volatile Compounds of GF Cakes

The volatile profile of GF cakes consisted of 29 compounds, which were classified into aldehydes, alkanes, alkenes, alcohols, aromatic hydrocarbons, acids, esters, furans, ketones, pyrazines, sulfur compounds, and terpenes. The control GF cake contained 20 volatile compounds, whereas the incorporation of LP and DFLP increased both the diversity and abundance of volatiles. Among all identified groups, aldehydes represented the dominant class of volatile compounds. The highest total aldehyde concentration was observed in sample C20 (127.83 mg/kg), followed by C10 (102.09 mg/kg), C15 (99.96 mg/kg), DFC20 (89.79 mg/kg), and DFC15 (75.49 mg/kg) (*p* < 0.05). The total aldehyde levels of control and DFC10 samples did not differ significantly (*p* > 0.05) (Table 7).

**TABLE 7:** Volatile compounds of GF cakes with LP and DFLP (mg/kg).^1^ ^,^ ^2^

Kovats index	Volatile compound	Sensory definition^3^	Control	DFC10	DFC15	DFC20	C10	C15	C20
	*Aldehydes*								
506	Propanal	Sharp, fruity	0.82 ± 0.04^a^	8.87 ± 0.20^a^	1.40 ± 0.31^bc^	1.46 ± 0.14^bc^	1.00 ± 0.08^a^	1.13 ± 0.02^ab^	1.53 ± 0.07^c^
595	Butanal	Fruity, buttery	1.49 ± 0.12^b^	0.95 ± 0.03^a^	2.29 ± 0.21^c^	2.34 ± 0.16^c^	1.46 ± 0.18^b^	1.60 ± 0.12^b^	1.71 ± 0.06^b^
696	3‐Methylbutanal	Hazelnut, fruity, malty	4.13 ± 0.07^a^	8.74 ± 0.91^b^	15.04 ± 0.87^c^	19.29 ± 0.99^d^	14.08 ± 0.14^c^	19.82 ± 0.89^d^	34.21 ± 1.49^e^
706	2‐Methylbutanal	Fruity, nutty, malty	6.07 ± 0.12^a^	6.47 ± 1.23^ab^	12.24 ± 4.33^bc^	11.43 ± 3.94^abc^	11.21 ± 0.37^abc^	14.22 ± 0.93^c^	27.78 ± 0.95^d^
781	3‐Methyl‐2‐butenal	Sour, fruity	1.59 ± 0.09^bc^	0.87 ± 0.03^a^	1.44 ± 0.10^b^	2.41 ± 0.24^de^	2.07 ± 0.17^cd^	2.62 ± 0.48^e^	3.81 ± 0.04^f^
801	Hexanal	Green leaf, green beans	6.86 ± 0.48^a^	12.34 ± 0.50^b^	16.36 ± 0.22^c^	19.32 ± 0.95^d^	29.99 ± 1.10^e^	32.33 ± 0.81^f^	32.81 ± 0.93^f^
902	Heptanal	Fatty, citrus	5.08 ± 0.14^d^	1.67 ± 0.09^a^	1.49 ± 0.39^a^	1.07 ± 0.17^a^	3.42 ± 0.54^c^	2.80 ± 0.06^bc^	2.53 ± 0.06^b^
965	Benzaldehyde	Almond, sweet	11.60 ± 0.81^d^	3.42 ± 0.36^a^	5.63 ± 0.56^ab^	7.21 ± 3.23^bc^	10.25 ± 0.41^cd^	9.13 ± 0.86^cd^	5.30 ± 0.42^ab^
1049	Benzeneacetaldehyde	Floral, honey	3.46 ± 1.29^f^	2.85 ± 0.10^a^	5.46 ± 1.20^bc^	7.47 ± 1.34^cd^	23.09 ± 0.94^e^	9.15 ± 0.60^d^	4.80 ± 0.49^ab^
1107	Nonanal	Floral, fatty, bready	5.16 ± 0.11^a^	8.02 ± 0.41^b^	14.16 ± 0.91^c^	17.79 ± 1.14^d^	5.52 ± 0.38^a^	7.18 ± 0.64^b^	13.35 ± 0.51^c^
	Total		46.25 ± 2.04^a^	46.18 ± 3.10^a^	75.49 ± 6.66^b^	89.79 ± 7.80^c^	102.09 ± 2.05^d^	99.96 ± 2.43^cd^	127.83 ± 4.89^e^
	*Alkane*								
598	3‐Methylpentane	Gasoline, solvent	N.D.	1.53 ± 0.25^a^	2.16 ± 0.36^b^	3.31 ± 0.44^c^	N.D.	N.D.	N.D.
600	Hexane	Solvent, gasoline	4.44 ± 0.11^d^	2.53 ± 0.01^bc^	2.58 ± 0.30^bc^	2.96 ± 0.27^c^	2.91 ± 0.06^c^	2.16 ± 0.21^ab^	1.90 ± 0.03^a^
617	Pentane, 2,2‐dimethyl—	Sweet, chloroform	4.50 ± 0.12^c^	2.49 ± 0.16^a^	3.50 ± 0.29^b^	3.57 ± 0.06^b^	3.65 ± 0.42^b^	3.53 ± 0.36^b^	3.15 ± 0.13^b^
800	Octane	Gasoline, fatty	N.D.	4.30 ± 0.72^a^	4.91 ± 0.16^ab^	5.37 ± 0.17^b^	N.D.	N.D.	N.D.
	Total		8.94 ± 0.23^c^	10.85 ± 1.14^d^	13.15 ± 0.08^e^	15.22 ± 0.83^f^	6.56 ± 0.36^b^	5.69 ± 0.15^ab^	5.05 ± 0.16^a^
	*Alkene*								
895	2,6‐Dimethyl‐1‐heptene	Neutral, solvent	11.23 ± 0.97^e^	4.38 ± 0.36^b^	3.59 ± 0.35^b^	0.77 ± 0.17^a^	7.25 ± 0.36^d^	5.73 ± 0.40^c^	4.54 ± 0.32^b^
	Total		11.23 ± 0.97^e^	4.38 ± 0.36^b^	3.59 ± 0.35^b^	0.77 ± 0.17^a^	7.25 ± 0.36^d^	5.73 ± 0.40^c^	4.54 ± 0.32^b^
	*Alcohols*								
1037	2‐Ethyl‐1‐hexanol	Floral, green, waxy	N.D.	N.D.	1.45 ± 0.05^a^	2.47 ± 0.12^b^	N.D.	N.D.	N.D.
	Total		N.D.	N.D.	1.45 ± 0.05^a^	2.47 ± 0.12^b^	N.D.	N.D.	N.D.
	*Aromatic hydrocarbon*								
773	Toluene	Sweet, solvent	15.13 ± 0.24^e^	7.15 ± 0.07^a^	10.65 ± 0.54^bc^	12.79 ± 0.40^d^	12.09 ± 0.71^cd^	10.92 ± 1.18^bc^	9.98 ± 0.18^b^
	Total		15.13 ± 0.24^e^	7.15 ± 0.07^a^	10.65 ± 0.54^bc^	12.79 ± 0.40^d^	12.09 ± 0.71^cd^	10.92 ± 1.18^bc^	9.98 ± 0.18^b^
	*Acids*								
612	Acetic acid	Sour, sharp vinegar	N.D.	N.D.	N.D.	N.D.	N.D.	0.17 ± 0.02^a^	0.50 ± 0.03^b^
	Total		N.D.	N.D.	N.D.	N.D.	N.D.	0.17 ± 0.02^a^	0.50 ± 0.03^b^
	*Ester*								
1167	Acetic acid, phenylmethyl ester	Fruity, floral, sweet	N.D.	N.D.	N.D.	3.16 ± 0.07	N.D.	N.D.	N.D.
	Total		N.D.	N.D.	N.D.	3.16 ± 0.07	N.D.	N.D.	N.D.
	*Furans*								
614	2‐Methylfuran	Burnt, woody	3.06 ± 0.13^bcd^	1.60 ± 0.19^a^	2.64 ± 0.18^b^	3.41 ± 0.06^cd^	2.78 ± 0.21^bc^	3.57 ± 0.59^d^	4.86 ± 0.41^e^
832	2‐Furancarboxaldehyde	Sweet, caramel	2.87 ± 0.07^c^	1.94 ± 0.10^b^	1.18 ± 0.18^a^	0.87 ± 0.10^a^	2.74 ± 0.26^c^	1.22 ± 0.17^a^	1.11 ± 0.02^a^
882	2‐Furanmethanol	Sweet, burnt	28.93 ± 1.10^f^	15.44 ± 1.20^d^	8.20 ± 0.54^c^	1.14 ± 0.13^a^	18.33 ± 0.68^e^	7.85 ± 0.42^c^	4.84 ± 0.88^b^
993	2‐Pentylfuran	Green, fatty	16.19 ± 1.04^d^	15.12 ± 0.45^cd^	9.13 ± 0.60^b^	5.39 ± 0.19^a^	13.57 ± 0.78^c^	8.26 ± 0.67^b^	6.44 ± 0.53^a^
	Total		51.06 ± 1.94^f^	34.10 ± 0.66^d^	21.14 ± 0.07^c^	10.81 ± 0.48^a^	37.42 ± 1.52^e^	20.89 ± 1.84^c^	17.26 ± 1.85^b^
	*Keton*								
730	3,3‐Dimethyl‐2‐butanone	Sweet, solvent	N.D.	0.89 ± 0.15^a^	1.19 ± 0.09^b^	1.84 ± 0.19^c^	0.72 ± 0.09^a^	1.41 ± 0.01^b^	1.85 ± 0.14^c^
	Total		N.D.	0.89 ± 0.15^a^	1.19 ± 0.09^b^	1.84 ± 0.19^c^	0.72 ± 0.09^a^	1.41 ± 0.01^b^	1.85 ± 0.14^c^
	*Pyrazines*								
735	Pyrazine	Roasted, nutty, soil, coffee	N.D.	0.51 ± 0.01^a^	0.51 ± 0.00^a^	0.54 ± 0.07^a^	N.D.	N.D.	N.D.
828	Methylpyrazine	Roasted, nutty	N.D.	1.54 ± 0.44^ab^	1.84 ± 0.10^b^	3.70 ± 0.14^d^	1.07 ± 0.16^a^	2.61 ± 0.07^c^	4.12 ± 0.19^d^
	Total		N.D.	2.05 ± 0.45^b^	2.35 ± 0.10^bc^	4.24 ± 0.21^d^	1.07 ± 0.16^a^	2.61 ± 0.07^c^	4.12 ± 0.19^d^
	*Sulfur compound*								
515	Dimethyl sulfide	Sulfurous, boiled corn, cabbage	0.41 ± 0.03^a^	0.45 ± 0.04^a^	0.54 ± 0.02^b^	0.56 ± 0.01^b^	0.40 ± 0.07^a^	0.39 ± 0.02^a^	0.41 ± 0.01^a^
736	Dimethyl disulfide	Garlic, rotten cabbage	N.D.	0.75 ± 0.13^a^	0.87 ± 0.03^ab^	1.12 ± 0.01^c^	0.80 ± 0.07^a^	0.99 ± 0.05^bc^	1.06 ± 0.04^c^
	Total		0.41 ± 0.03^a^	1.20 ± 0.10^b^	1.41 ± 0.06^c^	1.68 ± 0.02^d^	1.20 ± 0.15^b^	1.38 ± 0.07^bc^	1.47 ± 0.03^c^
	*Terpenes*								
1033	dl‐Limonene	Citrus, fresh lemon	7.25 ± 0.36^bc^	5.42 ± 0.29^a^	6.93 ± 0.33^b^	7.90 ± 0.35^cd^	7.92 ± 0.05^cd^	8.18 ± 0.21^d^	9.72 ± 0.37^e^
	Total		7.25 ± 0.36^bc^	5.42 ± 0.29^a^	6.93 ± 0.33^b^	7.90 ± 0.35^cd^	7.92 ± 0.05^cd^	8.18 ± 0.21^d^	9.72 ± 0.37^e^

Abbreviation: N.D., not detected.

^1^ The analysis results were given as arithmetic mean ± standard deviation.

^2^ Different letters indicate statistical differences within row (*p* < 0.05).

^3^ Belitz et al. (2009), Garvey et al. (2021), and The Good Scents Company (2025).

The predominant aldehydes were 3‐methylbutanal, 2‐methylbutanal, hexanal, and nonanal, whose concentrations increased proportionally with the level of LP supplementation (*p* < 0.05). GF cakes formulated with DFLP exhibited significantly lower levels of 3‐methylbutanal, 2‐methylbutanal, and hexanal compared to GF cakes with LP (*p* < 0.05). In accordance with the present findings, Krause et al. (2022) also identified aldehydes such as hexanal, 2‐methylbutanal, and 3‐methylbutanal in cake samples. Strecker aldehydes are formed through reactions between amino acids and α‐dicarbonyl intermediates, including deoxyosones produced during the Maillard reaction. Specifically, 2‐methylbutanal and 3‐methylbutanal arise from the oxidative decarboxylation of isoleucine and leucine, respectively (Belitz et al. 2009). These compounds are typically characterized by pleasant malty and sweet aroma notes (Garvey et al. 2021). Hexanal is a key indicator of linoleic acid oxidation and contributes to grassy or beany odor attributes (Belitz et al. 2009). Nonanal, another lipid oxidation product, is associated with bready and cake crust‐like sensory notes (Garvey et al. 2021). The higher levels of these volatile compounds in GF cakes enriched with LP, compared to GF cakes with DFLP, are likely due to the higher lipid content of LP, which enhances the formation of lipid‐derived aldehydes.

Furans constituted the second most abundant class of volatile compounds following aldehydes. The addition of LP or DFLP led to a significant decline in the concentrations of furan derivatives (*p* < 0.05). In particular, the levels of 2‐furancarboxaldehyde, 2‐furanmethanol, and 2‐pentylfuran were markedly lower in LP supplemented cakes than in the control (*p* < 0.05). Moreover, increasing the proportion of LP resulted in a progressive decrease in these furan compounds (*p* < 0.05). Furan‐derived volatiles are mainly generated through the dehydration and fragmentation of sugars during Maillard‐type reactions or via caramelization processes resulting from the direct thermal degradation of sugar molecules (Zhang et al. 2012). These compounds are typically associated with characteristic sensory notes described as earthy, caramel‐like, and biscuit‐like aromas (Matsakidou et al. 2010). Consistent with the findings of the present study, Wieczorek et al. (2022) also reported a decrease in the concentration of furan‐derived volatile compounds in breads enriched with cricket powder.

Neither pyrazine nor methylpyrazine was detected in the control GF cake. Pyrazine was identified exclusively in DFC10, DFC15, and DFC20 samples, while methylpyrazine was most abundant in DFC20 and C20 (*p* < 0.05). The formation of these Maillard‐derived compounds suggests that LP, particularly in its non‐defatted form, may enhance thermal reactions during baking, thereby influencing the overall volatile profile of the GF cakes. Pyrazines are typically formed through Maillard reactions involving amino acids and reducing sugars during thermal processing. Their absence in the control cake and appearance in LP and DFLP containing cakes suggest that locust flour supplied additional amino acid precursors that promoted pyrazine formation during baking. The higher pyrazine concentrations observed at increased substitution levels further support the role of insect‐derived proteins in the generation of roasted and nutty aroma compounds. Similarly, the incorporation of locusts (*Ruspolia differens*) in cookies (Ochieng et al. 2023) and crickets in bread (Wieczorek et al. 2022) was found to increase pyrazine content.

Sulfur‐containing compounds also increased following LP and DFLP incorporation (*p* < 0.05). The formation of dimethyl sulfide and dimethyl disulfide may be associated with the thermal degradation of sulfur‐containing amino acids, particularly methionine and cysteine, which act as important precursors of sulfur volatiles during thermal processing (Luo et al. 2024). Although detected at relatively low concentrations, sulfur compounds possess very low odor thresholds and may therefore contribute substantially to the overall aroma profile of baked products. The concentration of limonene increased in LP containing cakes, particularly at higher substitution levels (*p* < 0.05). This finding may indicate the contribution of volatile constituents naturally associated with locust flour or differences in volatile retention within the cake matrix following insect flour incorporation.

### 3.7 Microbiological Analysis

Microbiological evaluation of food is a crucial area for understanding the suitability of food for human consumption. In 2015, the European Food Safety Authority (EFSA) published a scientific opinion on the risks of producing and consuming insects as food and feed. This opinion stated that edible insects could be considered an alternative protein source when appropriate production and processing conditions are provided; however, microbiological, chemical, and allergenic risks should be carefully evaluated depending on the substrate and production method used (EFSA 2015). For this purpose, microbiological analyses are important in foods produced with insects. In this study, no pathogens, yeasts, or molds were detected in any of the GF cakes containing control, LP, and DFLP (Table 8). A statistically significant difference was found between the control sample and the GF cakes with added LP and DFLP in terms of total aerobic mesophilic bacteria count (*p* < 0.05). The highest total aerobic mesophilic bacteria count was determined in the DFC20 sample (2.01 cfu/g), while the lowest count was observed in the control sample (1.23 cfu/g). Adding up to 15% LP or DFLP to GF cakes did not create a statistically significant difference in the total aerobic mesophilic bacteria count of the samples (*p* > 0.05).

**TABLE 8:** Microbiological properties of GF cakes (cfu/g).

Sample	Total aerobic mesophilic bacteria	Yeast and molds	Pathogens^1^
Control	1.23 ± 0.03^a^	N.D.	N.D.
C10	1.79 ± 0.05^b^	N.D.	N.D.
C15	1.83 ± 0.07^b^	N.D.	N.D.
C20	1.96 ± 0.02^cd^	N.D.	N.D.
DFC10	1.81 ± 0.08^b^	N.D.	N.D.
DFC15	1.87 ± 0.05^bc^	N.D.	N.D.
DFC20	2.01 ± 0.09^d^	N.D.	N.D.

Abbreviation: N.D., not detected.

^1^ Pathogens: *Escherichia coli, Bacillus cereus, Staphylococcus aureus*.

The number of bacteria, yeasts, and molds is important indicators of the shelf life of foods containing insects such as locust (Bernaert et al. 2022). In a study by Kowalski, Mikulec, et al. (2022) in which yellow mealworm ( *Tenebrio molitor* ) was added to sponge cakes, no pathogens, yeasts, molds, or total aerobic mesophilic bacteria were detected in the sponge cakes. A slight increase in the number of molds, yeasts, and bacteria was observed after 30 days of storage of the cakes. It was concluded that low moisture content and high baking temperature suppress the growth of pathogenic microflora in food (Kowalski, Mikulec, et al. 2022). In a study where mealworms ( *T. molitor* ) were added to bread, it was reported that no yeast or mold was found in any of the breads. At the same time, the total number of viable bacteria was found to be quite low. No significant difference was found in total viable bacterial counts among bread samples enriched with insect powder (*p* > 0.05) (Gantner et al. 2022). In a study by Roncolini et al. (2019), an insignificant amount of aerobic bacterial spores was detected in bread crumbs to which mealworms were added. It was reported that this result may be due to the heat resistance of the spores because the heat center of the bread was baked below 100°C (Roncolini et al. 2019).

### 3.8 Sensory Analysis

In the sensory evaluation, color, texture, flavor, and overall acceptability of the GF cake samples were assessed (Table 9). The color of the GF cakes was positively influenced by the addition of both LP and DFLP, with the C20 sample receiving the highest color preference from the panelists (*p* < 0.05). The use of LP contributed to the improvement of texture characteristics, and the C15 sample exhibited superior texture properties compared with the other formulations (*p* < 0.05). No statistically significant differences were observed among the C10, C20, and DFC15 samples in terms of texture attributes (*p* > 0.05). The highest texture score observed in samples with moderate LP substitution (particularly C15) suggests that intermediate incorporation levels provide an optimal balance between structural reinforcement and matrix disruption in GF cakes. At this level, the increased protein content from locust flour may enhance the cake structure by improving the interaction between starch and protein networks, leading to a more cohesive crumb. In contrast, higher substitution levels or the use of defatted formulations may disturb the continuity of the starch‐based matrix and reduce structural homogeneity, which negatively affects sensory mouthfeel. The incorporation of DFLP enhanced the flavor properties more than LP (*p* < 0.05). The DFC10 sample received the highest flavor scores, while the C20 was the least preferred in terms of flavor (*p* < 0.05). Regarding overall acceptability, no significant differences were found among the C10, C15, DFC10, and DFC15 samples and all these samples were rated higher than the control (*p* < 0.05).

**TABLE 9:** Sensory properties of GF cakes.^1^ ^,^ ^2^

Sample	Color	Texture	Flavor	Overall acceptability
Control	6.33 ± 0.62^a^	7.27 ± 0.70^b^	7.13 ± 0.52^ab^	7.33 ± 0.49^a^
C10	7.53 ± 0.52^cd^	7.67 ± 0.49^bc^	7.87 ± 0.64^cd^	8.13 ± 0.64^b^
C15	7.93 ± 0.46^de^	7.87 ± 0.64^c^	7.33 ± 0.62^ab^	8.40 ± 0.51^b^
C20	8.20 ± 0.68^e^	7.40 ± 0.51^bc^	7.07 ± 0.70^a^	7.20 ± 0.56^a^
DFC10	7.07 ± 0.70^b^	7.33 ± 0.62^b^	8.20 ± 0.56^d^	8.20 ± 0.41^b^
DFC15	7.47 ± 0.52^bc^	7.60 ± 0.74^bc^	7.93 ± 0.46^cd^	8.07 ± 0.59^b^
DFC20	7.93 ± 0.59^de^	6.67 ± 0.49^a^	7.53 ± 0.52^bc^	6.93 ± 0.59^a^

^1^ The analysis results were given as arithmetic mean±standard deviation.

^2^ Different letters indicate statistical differences within column (*p* < 0.05).

**FIGURE 1:**
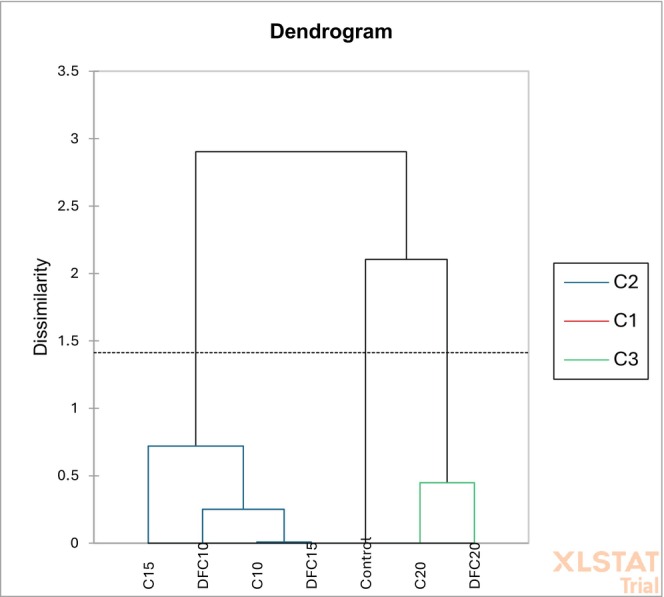
Dendrogram of GF cakes.

**FIGURE 2:**
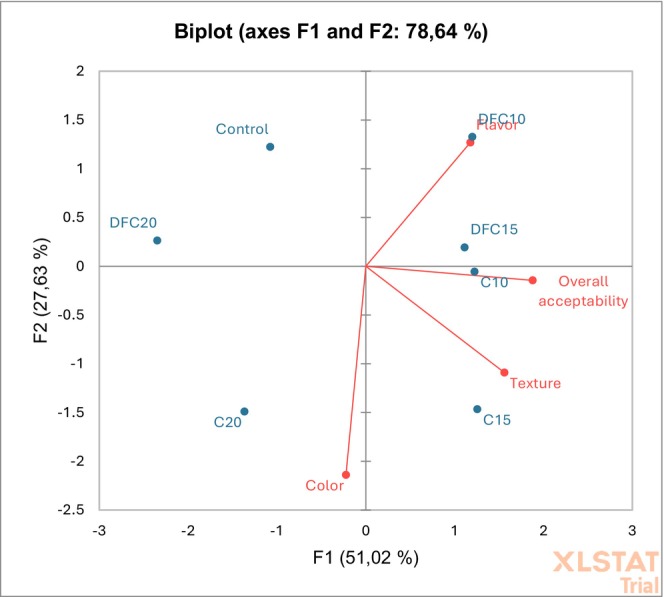
Principal component analysis for sensory properties of GF cakes.

Cluster Analysis and Principal Component Analysis (PCA) were applied using sensory analysis results to evaluate the relationship among the GF cakes. C10, C15, DFC10, and DFC15 samples formed the first group, the control sample constituted the second group, and C20 and DFC20 samples comprised the third group (Figure 1). PCA was applied to identify the sensory attributes that contributed to the formation of these groups. According to the PCA results, F1 accounted for 51.02% of the total variance, while F2 explained 27.63%. Texture, flavor, and overall acceptability were influential in the differentiation of C10, C15, DFC10, and DFC15 samples, whereas color was identified as the main parameter responsible for distinguishing the C20 and DFC20 samples (Figure 2). The sensory clustering results support these findings, indicating that moderate substitution levels formed a distinct group characterized by higher overall acceptability. PCA analysis further revealed that texture, flavor, and overall acceptability were the primary drivers of sample differentiation, whereas color contributed mainly to the separation of high‐substitution samples such as C20 and DFC20.

## 4 Conclusion

The locust (*L. migratoria*) can be considered a novel and sustainable alternative protein source for the food industry. The replacement of rice flour with LP and DFLP in GF cake formulations enhanced nutritional quality and supported sustainable food production. The results demonstrated that GF cakes containing LP and DFLP exhibited higher ash, protein, TPC, antioxidant activity, baking loss, and uniformity index values compared to the control sample, while specific volume and symmetry index values were reduced. The incorporation of LP and DFLP increased both the diversity and concentration of volatile compounds and improved the essential amino acid and essential fatty acid profiles of GF cakes. Sensory evaluation indicated that LP and DFLP positively influenced color and overall acceptability compared to the control, while DFLP was more effective than LP in enhancing flavor attributes. Overall, the findings suggest that locust‐based ingredients have potential for developing value‐added, protein‐enriched GF bakery products. This study contributes to the growing body of research on alternative protein sources aimed at addressing increasing food demand associated with population growth. The use of *L. migratoria* may offer promising opportunities for future research and product development, particularly in the formulation of nutritionally enhanced foods for individuals adhering to a GF diet.

## Author Contributions

**Yağmur Özcan:** investigation, formal analysis, writing – original draft, writing – review and editing, methodology, data curation. **Ceyda Dadalı:** conceptualization, methodology, investigation, funding acquisition, writing – review and editing, writing – original draft, project administration, formal analysis, supervision, data curation.

## Funding

This study was supported by Ege University Research Foundation (FM‐GAP‐2024‐32456).

## Conflicts of Interest

The authors declare no conflicts of interest.

## Data Availability

The data that support the findings of this study are available from the corresponding author upon reasonable request.
